# Mechanical Behavior of Plasma-Treated Metal–Rubber Assemblies

**DOI:** 10.3390/molecules29235590

**Published:** 2024-11-26

**Authors:** Lazhar Benyahia, Marisol Ji, Fabienne Poncin-Epaillard

**Affiliations:** Institut des Molécules et Matériaux du Mans, IMMM, UMR CNRS n° 6283, Le Mans Université, Avenue Olivier Messiaen, 72085 Le Mans, France

**Keywords:** metal–elastomer assembly, tack, adhesion, mechanism, plasma coating

## Abstract

Metal–elastomer assemblies, such as aluminum–NBR and stainless steel–FKM, widely used for sealing or damping functions in various fields, are currently prepared with highly toxic bonding agents. To substitute the use of these liquids, plasma technologies were applied. The chemical nature of the plasma polymerized adhesives is found to have no influence on the viscoelastic properties of the elastomer. Furthermore, cohesive assemblies were prepared with acetylene, acrylic acid or maleic anhydride as plasma polymerized layers. Their adhesive performances were evaluated thanks to a tack-like test. Their adhesion mechanisms, even if complex, are namely identified as the interdiffusion of elastomer chains within the plasma-based polymer film and the thermodynamic adhesion. Specifically, we propose that the adhesiveness of metal–rubber assemblies, correlated to the maximum stress at failure in the tack-like test, is proportional to an energy per unit volume. This new variable is determined as the ratio of the surface tension to the thinness of the plasma adhesive.

## 1. Introduction

During the last decades, composites, a macroscopic combination of materials of different types and nature, have been used in many applications and industrial sectors, such as aeronautics [1], automotive, space [1,2], telephony, health monitoring, etc. [3]. This success is due to their light weight and increased performance thanks to new technological and fundamental developments [2,4]. Among these composites, polymer/metal joins stand out. However, considering the very different chemical nature of the two material classes, improving the interfaces and ensuring good adhesion remain a challenge today [2,5]. This requires an in-depth understanding of the adhesion mechanisms between polymers and rigid substrates [6].

Adhesion refers to several physicochemical phenomena occurring at the interface of two surfaces [7]. The main related mechanisms are mechanical interlocking, interdiffusion, chemical interactions and wetting. The mechanical interlocking characteristic of the mechanical attachment between two materials depends on different physical parameters like the viscosity of the adhesive, the roughness and the topography of the surfaces [8,9,10,11,12]. Diffusion by each macromolecule or chain segment at the interface enhances the adhesion of the assembly, especially in a solvated medium [13,14,15,16,17]. Indeed, the presence of a co-solvent enhances the diffusion of the polymer chains into the substrate, leading to a more powerful adhesive joint.

Different adhesion theories were proposed. The first theory concerns the chemical bond. Based on chemical reactivity at the surface of the elastomer/brass assemblies, Rae et al. [18] proposed that resultant chemical bonds can be stronger or weaker depending on their nature: interatomic (covalent, ionic and metallic) or intermolecular (hydrogen and Van der Waals).

This concept was also applied to the copper oxide and the epoxy function [19]. The second theory is known as thermodynamic adhesion or wetting and is the first criterion considered in the applications. It corresponds to the spreading behavior of a liquid over a surface, characterized by the contact angle of the liquid drop deposited on the surface [20,21]. This theory allows for determining the surface energy of each material as well as the energy or the work of adhesion between the two materials. A low interfacial energy enhances the assembly cohesion.

The assembly of an elastomer with a metallic substrate is rather difficult because of the almost opposite chemical and physical properties of each component. Their incompatibility is reduced in the presence of liquid adhesives as an intermediate layer, the so-called primary and secondary layer [22,23]. Adhesion primers are organic resins that can react with most metals, such as aluminum, steel, stainless steel, copper and brass, to form covalent bonds with the corresponding oxides. Secondary adhesive layers, often halogenated polymers compatible with the primary layer and the elastomer, diffuse into the elastomeric matrix and are involved in the elastomer’s crosslinking nodes [24]. The drawback of this process, besides the use of highly toxic liquids in general, is that it is based on a manual multi-step coating in the case of complex geometries and, therefore, non-homogeneous layers.

Plasma polymerization may solve this process inconvenient. It was applied successfully in many applications [25]. In the 1980s, a US patent [26] described radiofrequency or microwave plasma coating from sulfur precursors derived from cyclic thioethers (thiophene, tetrahydrothiophene and 2-ethylthiophene). Thus, the radicals resulting from the electronic impact on the carbon–sulfur bond of the precursor could participate in the elastomer vulcanization. Nevertheless, these precursors impose restrictive regulations according to Reach standards, and research has been shifted towards gaseous precursors such as acetylene [27,28], butadiene [28] and bearing sp2 carbons, potential sites of rubber attachment [29]. Plasma deposition on aluminum of a more polar precursor, maleic anhydride, followed by aminolysis, also improves the adhesion and peel resistance of an EPDM [30]. However, the result depends on the applied duty cycle that controls the concentration of alkyl groups and the crosslink density of the adhesive joint. Although chemical anchoring has been emphasized in previous works, the good mechanical strength of steel–NR assembly requires a minimum plasma poly(thiophene) thickness of 50 Å [31]. Another advantage of plasma technologies is that the plasma treatment can also act as pretreatment of the substrate, before the deposition. This could be a simple sputtering by Ar^+^ bombardment or a reactive erosion in the presence of hydrogen [28,29]. Such a pretreatment increases the metal roughness, reduces the oxide layer on the surface and helps to obtain, in the case of iron substrate for example, a reactive and iron-rich surface causing the formation of iron sulfide during vulcanization.

The vulcanization of elastomers generates inter- and intra-chain crosslinking of polymers. Depending on the progress of the reaction, a three-dimensional network is created, which results in a viscoelastic solid-like behavior. The origin of the elasticity is essentially entropic and is directly proportional to the density of elastically active chains [32]. Thanks to dynamic rheological measurements, it is possible to determine the viscous G″ and elastic G′ viscoelastic moduli and thus determine the liquid/solid transition of the elastomer during its crosslinking [33,34].

Various tests are commonly used to assess the strength of a bonded joint, mainly mechanical, such as peel or tack, for the most frequently used. In short, the joint is destroyed in a disassembly process, and the force or energy required to break the joint is measured [35]. In addition, the fracture surface has to be examined to deduce the fracture type where the limits are (i) adhesive if no deposit of the adhering material is found on one of the assembly substrates and (ii) cohesive if residues are found on both substrates. The tack test is commonly used to evaluate the tackiness of adhesive assembly, especially for pressure-sensitive adhesives [36], as well as elastomers-based joints [37,38]. This test is quite easy to perform because it needs only to bring a metal probe, in general, into contact with the polymer surface. The probe is then maintained in contact under a fixed time-controlled pressure. Then, the probe is removed, and the separation force is measured.

In previous studies [39,40], we showed that the pulsed plasma polymerization of three precursors (acetylene—Ac; acrylic acid—AA; maleic anhydride—MA) is a promising method for preparing model plasma layers of the metal–elastomer adhesion. In the present work, we focus on the behavior of the elastomer/metal assembly during a tack-like test, which is called tack hereafter. So, two types of deposition are carried out, either by pulsed (PW) or continuous waves (CW), which allows for functionalizing the layer or crosslinking it in the last case. Two-layer thicknesses are prepared: a very thin one of about 10 nm and a thicker one of up to 100 nm. Such an experimental path should combine the effects of chemical anchoring and interdiffusion with a chemically controlled surface and a relatively thick film. This is illustrated with aluminum–plasma deposit–NBR (Al–pp–NBR) and stainless steel–plasma deposit–FKM (SS–pp–FKM). We demonstrated that, for all compositions and assemblies elaborated in this work, the stress needed to break up the assembly is proportional to the ratio of the surface tension and the thinness of the plasma-based joint. This new variable could be considered as a new universal parameter that may characterize the effectiveness of the adhesive assembly.

## 2. Materials and Methods

### 2.1. Materials, Plasma Deposition and Assembly Preparation

#### 2.1.1. Materials

The organic precursors used without further purification were gaseous acetylene (Air Liquid, Orsay, France) and liquid acrylic acid (Sigma-Aldrich, Saint-Quentin-Fallavier, France), which were filled in a quartz tube connected to the chamber and vaporized due to the low pressure. The used solid maleic anhydride (Sigma-Aldrich) was filled in the quartz tube heated at 52 °C and vaporized in a gas line heated at 75 °C. The elastomers chosen were poly(acrylonitrile butadiene) rubber (NBR) containing a PVC fraction (17%) provided by Safran Society and a vinylidene fluoride-based (FKM) elastomer. Two metallic substrates were used: anodized aluminum plates, type 7050, and low-carbon stainless steel, type 360 L. Both substrates were supplied by industrial partners. Substrates were machine-turned and polished. The average roughness Rz did not exceed 3 μm. The metallic substrates were cleaned in acetone and ethanol solutions and then plasma-treated in an argon atmosphere (*P* = 200 W, *Q_Ar_* = 20 sccm and *t* = 20 min) just before the plasma deposit.

#### 2.1.2. Plasma Process

Plasma polymerization was performed in a capacitive radio-frequency (RF, 13.56 MHz) plasma reactor. The low pressure was maintained thanks to a turbomolecular pump Alcatel ATP-80 (Alcatel-Lucent, Massy, France) coupled with a primary rotary pump Pfeiffer Vacuum Adixen 2015SD Pascal (Pfeiffer Vacuum SAS, Annecy, France). Typical residual pressures were maintained between 10^−3^ and 10^−2^ Pa, while the working pressure (*p*) was kept around 1 Pa and measured by a wide-range capacitive-penning pressure gauge Alcatel ACC 1009 (Alcatel-Lucent, Massy, France). The glow discharge was sustained between two parallel electrodes separated by a fixed distance of 12 cm and powered with a Cesar RF generator (Advanced Energy, Fort Collins, CO, USA), with powers (*P*) ranging from 5 to 100 W. Reflected power was kept minimum thanks to an RF Navio matchbox (Advanced Energy). Plasma discharges were sustained in different atmospheres: acetylene at different flow rates (*Q* from 10 to 40 sccm) and in a vapor of pure AA or MA. For the latter, as their flow rates were not controlled, their content in the chamber was fixed with a constant working pressure of 1 Pa. Two modes were used to polymerize the precursor at the substrate interface: a pulsed (PW, variable frequency *f* and duty cycle dc) or continuous (CW) one. Changing the time allows for modulating the thickness of the layer. Finally, four types of layers were prepared: a thin and functionalized layer (PW), a thin but crosslinked one (CW), a thin and surface-functionalized one (CW + PW), and another one thick and crosslinked (CW × 2). The plasma parameters for the different precursors and model layers are described in Table 1.

#### 2.1.3. Vulcanization Process

The vulcanization kinetics was followed by rheometry using ARESG2, (TA Instruments, Guyancourt, France) with a plan/plan (diameter = 8 mm) geometry in a thermostatically controlled oven. Then, the vulcanization started with a rapid heating up to the vulcanization temperature (160 °C). At 150 °C, a compression stress of 0.3 MPa was applied to enhance the contact between the plasma polymer layer and the elastomer and avoid the elastomer foaming. The viscoelastic shear moduli, elastic (G′) and viscous (G″), were measured at a frequency of 10 rad/s and a strain of 0.1%. The vulcanization was maintained during 850 s. Initially, the thickness of the elastomer was 3.5 ± 0.3 mm, but when the compression was applied, the thickness reduced up to 0.5 mm. Finally, the system was cooled to 30 °C while following the G′ and G″ modulus without applying any axial force by the rheometer.

### 2.2. Assembly Decohesion Followed by Tack-like Measurement

The assembly strength was evaluated using tack-like measurements, called tack hereafter. In practice, this test is closely related to a standard method called ASTM D429 Type A (https://www.astm.org/d0429-14e01.html, accessed on 24 July 2024), a standard method test for rubber property adhesion to rigid substrates. The assembly was prepared with the elastomer (thickness = 3.5 ± 0.3 mm, diameter = 8.0 mm) inserted between two metallic disks (aluminum, diameter = 8 mm). Beforehand, the latter were cleaned with acetone in an ultrasonic bath and plasma-treated in an argon atmosphere (*P* = 200 W, *Q_Ar_* = 20 sccm and *t* = 20 min); they were layered with the plasma coating polymer. The same disks were used for the rheological measurements. The strength needed for assembly decohesion was measured with the tensile machine ZwickRoell Z0101 (ZwickRoell, Ars Laquenexy, France) equipped with a strength sensor Xforce P 500N type, (ZwickRoell, Ars Laquenexy, France) with a 2 mV/V sensitivity. During the measurements, an axial displacement took place. The applied strength was measured versus the shift at a constant rate (0.1 mm/min) at room temperature comprised between 20 and 25 °C. The stress and deformation values were then extracted.

## 3. Results

### 3.1. Influence of the Plasma-Deposit Adhesive on Vulcanization Kinetics

The rheological monitoring during vulcanization allows for determining the viscoelastic shear moduli, G′ and G″. An example of the evolution of the viscoelastic moduli during vulcanization is shown in Figure S1 for Al–NBR assemblies and corresponds to a classical rheological evolution of an elastomer during vulcanization [41,42,43]. Nevertheless, the description focuses only on the time evolution of G′ during the curing. We distinguished four zones: Zone I corresponds to the softening of the elastomer at the beginning of heating; Zone II illustrates the initiation of crosslinking; Zone III is the accomplishment of the vulcanization during the isotherm stage; Zone IV corresponds to the cooling of the sample. Figure S2 presents the time evolution of G′ during curing for Al–NBR assemblies in the presence of the three pulsed-plasma polymers deposited with the optimized conditions (PW). Regardless of the nature of the plasma polymer, the time dependence of G′ is invariant within the four zones in comparison to the assembly without a plasma polymer. Moreover, the plateau values at the end of curing are close to each other. The small variation in the moduli during curing, around 10%, is within the acceptable limit of the reproducibility of the rheological measurements. This rheological monitoring, therefore, only concerns the crosslinking of the elastomer, as the plasma deposition does not influence the vulcanization kinetics or the modulus of the elastomer after vulcanization. Similar results are obtained with SS–pp–FKM assemblies with the different plasma treatments.

### 3.2. Mechanical Behavior of Al–pp–NBR and SS–pp–FKM Assemblies

#### 3.2.1. Tack Measurements

The mechanical strength of the assemblies is assessed by measuring the breaking out force (F) versus the displacement (d) in a tensile test. This test is similar to a tack test and is referred to as such in the following. Since the rupture force also depends on the elastomer deformation, the nominal stress (σ = $\frac{F}{S}$) as a function of true deformation (ε = $\ln(\;\frac{d+l}{l})$) [44] is rather considered. S and l are the initial area and length of the sample, respectively. d is the crosshead’s displacement of the tensile machine. Figure S3 shows typical curves of the tack test for all Al–pp–NBR prepared with PW plasma parameters. One can notice the good reproducibility of the measurements. Furthermore, at the beginning of the measurement, the stress increases mostly linearly with the strain, followed by a non-linear behavior before reaching maximum stress (σ_M_) related to the breakpoint of the set. Above this point, σ decreases until the total disassembly, mostly related to the decrease in contact area during the axial displacement. This behavior is similar to what was reported for a tack experiment and was related to cavitation appearing at the interface between the tool and the material [35]. However, the setup of the present experiment prevents this phenomenon from being observed.

#### 3.2.2. Influence of the Different Plasma Deposits on the Apparent Elastic Modulus and the Maximum Stress During Tack Measurements

At low deformations (<0.02%), during the linear behavior, the slope of stress (σ) versus deformation (ε) represents the apparent elastic modulus (E) of the material. For isotopic material, the elastic modulus (E_rheo_) can be deduced from the viscoelastic measurements knowing the shear moduli (G) and using the Poisson coefficient (υ): E_rheo_ = 2(1 + υ)G′. For incompressible sample υ = 0.50, so E_rheo_ = 3G′ [45,46], which reads to 10.40 MPa and 11.67 MPa for Al–pp–NBR and SS–pp–FKM with any plasma deposit, respectively, at room temperature. Therefore, if E is lower than E_rheo_, this implies that the elastomer is not sufficiently stretched during the elongation test because the assembly releases the stress before it increases sufficiently and deforms the elastomer.

Tables S1 and S2 show E and E_rheo_, respectively, for Al–pp–NBR and SS–pp–FKM obtained with different precursors and plasma treatments. With Al–pp–NBR assemblies coated with pp–Ac and pp–MA, E is higher than E_rheo_, which is not the case for pp–AA. This suggests that the last assembly is too weak to support the stress developed by the deformation of the elastomer. Moreover, just after vulcanization, the decohesion is observed with some assemblies sealed with pp–AA. It appears that the letter induces a worse reproducibility than other precursors. For each plasma polymer, repeatability is not as reliable, and consequently, plasma parameters may not have a significant influence. In the case of SS–pp–FKM assemblies sealed with pp–AA and pp–MA, E is higher than E_rheo_ but weaker for pp–Ac. This suggests that the last assembly is too weak to support the stress developed by the deformation of the elastomer and that the chemical nature of the precursor may play a role in the assembly decohesion.

**Figure 1:**
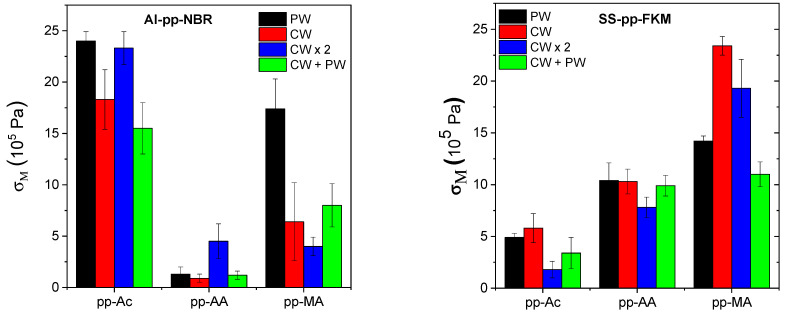
The maximum stress σ_M_ at the failure of the assemblies, Al–pp–NBR (**left**) and SS–pp–FKM (**right**), for different plasma deposits as indicated in the figure.

Figure 1 shows σ_M_ for Al–pp–NBR and SS–pp–FKM for different precursors and plasma treatments. For Al–pp–NBR, the Ac plasma polymer is the most efficient regardless of the deposition parameters. Pp–MA leads to lower failure stress levels than pp–Ac, but it is still much higher than pp–AA, especially in PW deposition conditions. As mentioned above, with some Al–pp–AA–NBR, assemblies are very weak, sometimes breaking even before their tack measurement in the tensile machine. In this case, the deduced apparent elastic modulus is much lower than that calculated from rheological measurements. This implies that the level of stress supported by the adhesive joint is much lower than the stress that would be developed to stretch the elastomer itself. For SS–pp–FKM, the best–performing precursor is MA; the worst-performing is Ac, but it still produces much higher levels of stress at failure than pp–AA with Al–pp–NBR. It should also be noted that there does not appear to be a systematic effect of deposition conditions on stress at failure for either Al–pp–NBR or SS–pp–FKM.

### 3.3. Correlation Between the Maximal Stress σ_M_ and the Model Plasma Deposit

In the following, the dependence of the threshold stress for failure initiation (σ_M_) on the physicochemical properties of the plasma deposit (thickness, interfacial energy and chemical composition) is studied. The plasma coating characterization was fully described in [39,40].

#### 3.3.1. Chemical Dependence of the Maximal Stress

The chemical anchoring between two organic materials is based on the creation of covalent bonds. In our previous work [39,40], we identified the chemical functions that are able to react with the elastomer, alkene (CC) and carbonyl (CO). Their respective and relative concentrations (ratio CC or CO/aliphatic CH) were quantified for the four model layers issued from the three selected precursors. The wavenumber area of the different FTIR vibration bands is given in Table S3.

**Figure 2:**
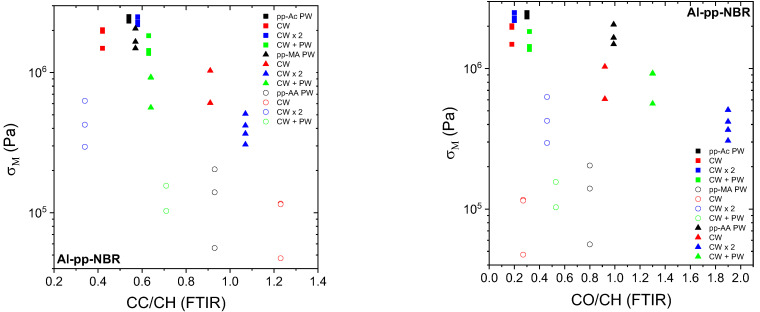
Dependence of the maximal stress σ_M_ on the chemistry determined by FTIR spectroscopy for Al–pp–NBR assemblies as indicated in the figure. Empty symbols correspond to assemblies for which E is lower than E_rheo_.

For the Al–pp–NBR assemblies, there is no specific dependence of σ_M_ on the alkene concentration, or at least a slight tendency to decrease with the CC/CH ratio. Even if the data are noisy (Figure 2 left), it is possible to observe a relative clustering according to the nature of the plasma polymer. For pp–Ac, σ_M_ remains close to each other, unlike pp–MA and pp–AA layers. The same remark could be drawn for the CO/CH ratio (Figure 2 right). The low carbonyl content of pp–Ac results from post-oxidation. It is worth noting that for some weak assemblies, the failure prematurely happens before an effective elongation of the elastomer (empty symbols in Figure 2). Thus, these values should therefore be considered with caution. In fact, what appears to be an exception to the trend described above, in the case of pp–MA, is merely the consequence of this behavior. However, we prefer to show all the data to give a complete picture of this system.

**Figure 3:**
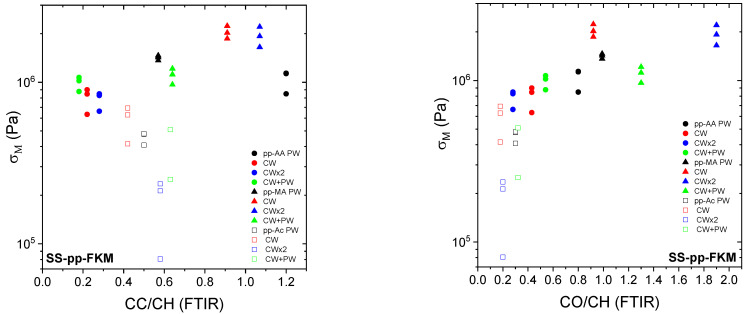
Dependence of the maximal stress σ_M_ on the chemistry determined by FTIR spectroscopy for SS–pp–FKM assemblies as indicated in the figure. Empty symbols correspond to assemblies for which E is lower than E_rheo_.

For the SS–pp–FKM assemblies, σ_M_ does not show a significant trend with the CC/CH ratio (Figure 3 left). When SS–pp–Ac–FKM data are ignored because of their lower E compared to E_rheo_, the stress appears to increase slightly, and so it is for the CO/CH ratio (Figure 3 right). This growing trend is even more marked for all plasma joints polar (pp–AA, pp–MA) and functionalized (PW, CW + PW), as noticed for pp–AA. But, for pp–MA coatings, it is more difficult to identify a relationship between the deposition mode and the assembly cohesion since, in any case, σ_M_ is increasing versus the carbonyl concentration. On the other hand, the higher functionality of pp–MA induces greater stress than that of pp–AA joints.

In summary, with Al–pp–NBR assemblies, the contribution of chemical functions seems to depend little on the chemical anchoring for their cohesion. In the case of SS–pp–FKM, chemistry seems to barely influence the mechanical properties of the assembly. This could be explained by the alkene reactivity of each part (joint and elastomer) and by chemical reactions through the electrophilic carboxylic acid or anhydride groups. A more detailed analysis of possible reaction mechanisms and the effect of the monomer’s nature on the plasma process can be found in [39,40].

#### 3.3.2. Thermodynamic Dependence of the Maximal Stress

The first criterion for adhesion is the good wetting between the plasma polymer and the elastomer. From the surface energies, dispersive ($\gamma_{1}^{d}$; $\gamma_{2}^{d}$) and polar ($\gamma_{1}^{p}$; $\gamma_{2}^{p}$) parts of each material, given in [40], the interfacial free energy γ_12_ [47] between the plasma polymer (1) and the elastomer (2) is determined as $\gamma_{12}=\gamma_{1}+\gamma_{2}-2\left(\sqrt{\gamma_{1}^{p}\gamma_{2}^{p}}+\sqrt{\gamma_{1}^{d}\gamma_{2}^{d}}\right)$.

**Figure 4:**
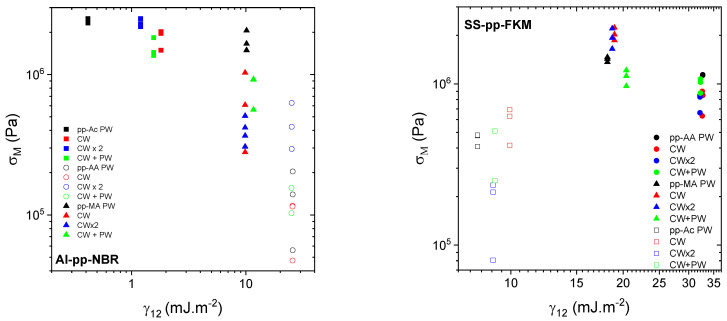
Dependence of the maximal stress σ_M_ on interfacial energy γ_12_ for Al–pp–NBR (**left**) and SS–pp–FKM (**right**) assemblies as indicated in the figure. Empty symbols correspond to assemblies for which E is lower than E_rheo_.

For Al–pp–NBR, σ_M_ gradually decreases with increasing the interfacial energy γ_12_ (Figure 4 left). This behavior seems to be linked to the chemical nature of the precursor. Clearly, pp–Ac adheres more strongly to NBR and metal than the other precursors. Furthermore, their low contact angle with water and their polar energy vary little depending on plasma conditions [40].

Figure 4 shows the variation in maximal stress as a function of the plasma polymer/elastomer interfacial energy for SS–pp–FKM assemblies. Unlike with Al–pp–NBR assemblies, the range of γ_12_ variations goes as high as ≈33 mJ·m^−2^, but σ_M_ of assemblies is higher with pp–MA but decreases when pp–AA is used. The latter has a higher γ_12_, meaning that adhesion is unfavorable as the value of γ_12_ increases.

Finally, the overall shape of this curve indicates that thermodynamic aspects play a role in the assembly cohesion.

#### 3.3.3. Thickness Dependence of the Maximal Stress

**Figure 5:**
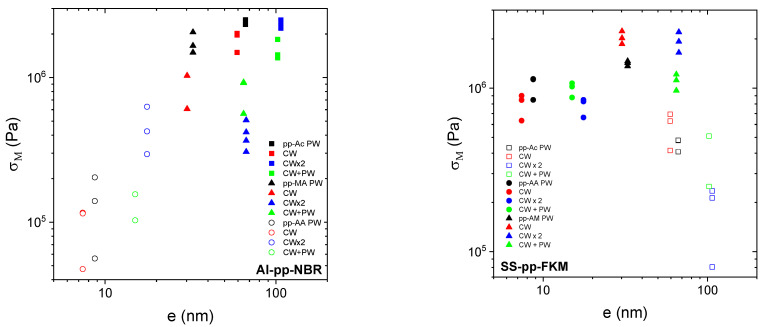
Dependence of the maximal stress σ_M_ on plasma joint thickness for Al–pp–NBR (**left**) and SS–pp–FKM (**right**) assemblies as indicated in the figure. Empty symbols correspond to assemblies for which E is lower than E_rheo_.

One of the adhesion mechanisms deals with the interdiffusion promoting the creation of interphase. To that end, we study the influence of the thickness of plasma polymer films on the adhesion behavior considering the maximum stress σ_M_. For all Al–pp–NBR assemblies, σ_M_ varies from 0.05 MPa to 2.5 MPa (Figure 5 left). It increases with the thickness layer, with a weaker rate for thicker films than 60 nm as pp–Ac films. These films also present an apparent elastic modulus higher than the corresponding elastomer one. However, the nature and the deposition mode seem to counteract the effect of the thickness. Indeed, the films with the same thickness (≈60 nm) but prepared from Ac in PW or CW conditions and from MA in CW × 2 or CW + PW conditions lead to different σ_M_. This conclusion can be contrasted with published results on the effect of the thickness on the cohesive failure strength in a single lap joint [48]. Simulations and comparison with some experimental results showed that the failure strength decreases with the thickness of the adhesive. However, it should be noted that the thicknesses in the two studies are remarkably different, some mm compared with a few nm in this work. We believe that the results of [48] put more emphasis on the mechanical contribution of the adhesive film thickness than on the molecular layer responsible for the adhesive bonds between the elastomer and the metal.

Therefore, one may conclude that the diffusion phenomenon is not unique, but other factors, like chemical or thermodynamics, may also account for Al–pp–NBR cohesion.

With SS–pp–FKM assemblies (Figure 5 right), σ_M_ increases from 0.6 MPa to 2.2 MPa with the increase in the adhesive joint thickness regardless of the results obtained with pp–Ac. Indeed, these assemblies have an apparent elastic modulus lower than the FKM one. Regarding the experimental results, two behaviors related to the chemical nature of the seal appear, namely, the deposits of pp–AA, on the one hand, and those of pp–AM, on the other hand. In addition, for the same thickness, two stress levels are observed. For pp–AA, the highest σ_M_, of about 1.1 MPa, is observed for the PW and CW + PW deposits. On the contrary, the lowest σ_M_, of about 0.6 MPa, is observed for CW and CW × 2 conditions. Despite the different thicknesses, the needed stress to separate the assembly remains the same for pp–AA. This phenomenon is also observed for pp–AM coatings with σ_M_ varying from 0.9 to 2.2 MPa. On the other hand, the highest σ_M_ is reached with continuous plasma treatment while the lowest σ_M_ is obtained with the pulsed one. Despite the noise in the measurements, it appears the limit adhesion stress uniformly increases with the thickness, which, at this stage, can be qualified as inconsistent. However, it is difficult to interpret the dependence of the stress on the deposition mode.

## 4. Discussion

To elucidate the mechanisms responsible for the adhesion of the joint and its performance, it is necessary to consider the interaction forces and the diffusion phenomenon at the scale of the plasma joint thickness. Two approaches are then proposed: (i) at the molecular scale, by considering specific bonds with the two studied assemblies, and (ii) in a more macroscopic way, by integrating all the interaction forces under the concept of interfacial tension. These two quantities are then modulated by the thickness of the plasma joint at different scales. At the molecular level, the quantity of specific bonds is integrated by multiplying by the thickness of the plasma polymer film. At the macroscopic scale, we suggest introducing an energy per unit volume given as the ratio of the interfacial tension γ_12_ by the thickness of the plasma seal.

On a molecular scale, σ_M_ is plotted as a function of the integrated reactive functions of the pp coating, determined by FTIR (CC/CH and CO/CH). The results for Al–pp–NBR and SS–pp–FKM assemblies are given in Figure S4 and Figure S5, respectively. Thus, the integration of the thickness into the consideration of specific bonds allowed for grouping all the points according to the nature of the monomer used to elaborate the pp joint for both Al and SS. For Al, σ_M_ seems to decrease as a function of the FTIR product (CC/CH and CO/CH) × thickness while it increases for SS. It is, therefore, not possible at this stage to conclude that the variation in this product has a systematic effect on the level of σ_M_. It seems that the consideration of other functions, such as hydroxyl, with a more specific FTIR signature, would be recommended.

**Figure 6:**
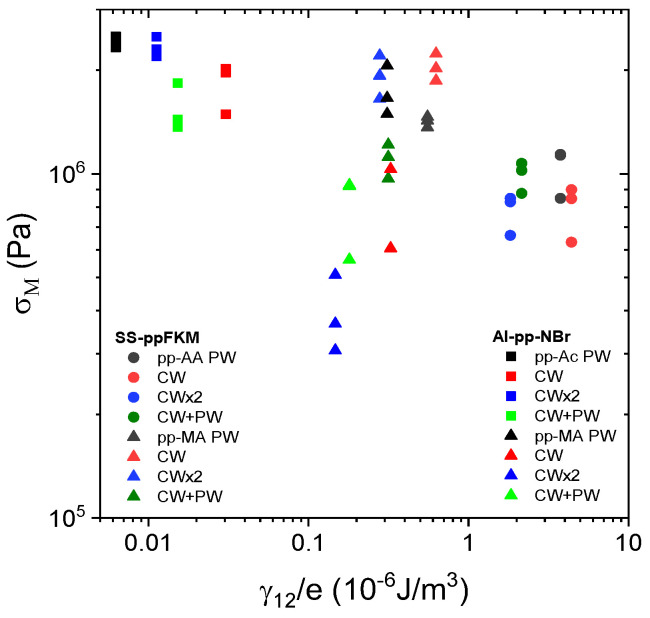
Dependence of the maximal stress σ_M_ on physical thermodynamics parameters (interfacial energy to thickness ratio, e/γ_12_) for Al–pp–NBR and SS–pp–FKM assemblies as indicated in the figure.

To this end, we propose to plot σ_M_ as a function of γ_12_/thickness. This result is given in Figure 6. As the analysis at the molecular level, this parameter confirms the importance of monomer chemistry. This is all the more remarkable as the data for the two joints cluster together. Moreover, the increase in this new parameter shows a very slight decrease in σ_M,_ which seems logical, i.e., the more this energy per unit of volume increases, the more the incompatibility of the pp seal with the elastomer increases. To confirm this trend, it would be very interesting to consider a test on larger samples and to consider a more reproducible test such as peeling, for example.

## 5. Conclusions

Plasma treatment was used to elaborate different polymer films acting as a primary adhesive joint to elaborate rubber/metal assemblies. For this purpose, continuous and pulsed plasma was used to create functionalized layers using acetylene, acrylic acid or anhydride maleic monomers. Then, rubber–metal assemblies, based on NBR–aluminum and FKM–SS, are elaborated. The vulcanization kinetics during the assembly were evaluated by measuring the viscoelastic moduli of the rubber during the curing. It follows that the rheological properties of the elastomer are independent of the plasma adhesive, whatever its nature.

The strength of the elastomer/metal-based substrate assemblies is then assessed with a tack test. More specifically, the maximum stress at the failure is correlated to different chemical and physical features of the plasma–polymer film. Particular attention is paid to the deconvolution of the three main factors: the interdiffusion of the elastomer chains within the plasma-based polymer film, the thermodynamic adhesion or the chemical adhesion.

The analysis has led to the suggestion of a new parameter, referred to as adhesion energy per unit volume, which is defined as the ratio of the interfacial tension to the thickness of plasma–polymer film. In fact, it allows us to propose a universal approach rationalizing the effect of chemical bonds and elastomer chain interdiffusion within the thickness of the plasma–polymer joint.

## Figures and Tables

**Table 1:** Plasma parameters applied to the different precursors depending on the model layer.

	PW	CW	CW × 2	CW + PW
pp–Ac *P* = 50 W, *Q* = 40 sccm	*f* = 7 kHz, *d.c.* = 11%, *t* = 60 min	*t* = 13 min	*t* = 26 min	CW: *t* = 13 min
				PW: *f* = 7 kHz, *d.c.* = 11%, *t* = 60 min
pp–AA *P* = 10 W, *p* = 1 Pa	*f* = 5 kHz, *d.c.* = 8%, *t* = 5 min	*t* = 2 min	*t* = 4 min	CW: *t* = 2 min
				PW: *f* = 5 kHz, *d.c.* = 8%, *t* = 5 min
pp–MA *P* = 10 W, *p* = 1 Pa	*f* = 5 kHz, *d.c.* = 8%, *t* = 20 min	*t* = 14.5 min	*t* = 29 min	CW: *t* = 14.5 min
				PW: *f* = 5 kHz, *d.c.* = 8%, *t*= 20 min

## Data Availability

Data are contained within the article.

## Supplementary Materials

The following supporting information can be downloaded at: https://www.mdpi.com/article/10.3390/molecules29235590/s1. Table S1: Young (tack) and Young (rheology) moduli for Al–pp–NBR for the different plasma deposits. Table S2: Young (tack) and Young (rheology) moduli for SS–pp–FKM for the different plasma deposits. Table S3: Wavenumber area of the different FTIR vibration bands. Figure S1: Time dependence of storage viscoelastic modulus G′ (black scares), loss viscoelastic modulus G″ (red circles) and temperature (blue triangles) during curing time for an Al–NBR assembly without plasma deposition. Figure S2: Time–dependence of the elastic modulus G′ during vulcanization for different Al–pp–NBR assemblies treated with a pulsed plasma (PW) with different precursors. Zone I corresponds to the softening of the elastomer at the beginning of heating, Zone II illustrates the initiation of crosslinking, Zone III is the accomplishment of the vulcanization during the isotherm stage. Zone IV corresponds to the cooling of the sample. Figure S3: Typical tensile curves showing the nominal stress σ versus the true strain ε for 3 Al–ppMA (PW)–NBR assemblies. The dashed line represent the linear fir of the beginning of the curve corresponding to the linear regime. The slop of this lie represents the Young modulus E. Figure S4: Dependence of the maximal stress σ_M_ on the chemistry determined by FTIR spectroscopy for Al–pp–NBR assemblies. Empty symbols correspond to assemblies for which E is lower than E_rheo_.Figure S5: Dependence of the maximal stress σ_M_ on the chemistry determined by FTIR spectroscopy for SS–pp–FKM assemblies. Empty symbols correspond to assemblies for which E is lower than E_rheo_.
