# Supplementary material for: Mechanical Behavior of Plasma-Treated Metal–Rubber Assemblies
Source: Molecules. 2024 Nov 26;29(23):5590. doi: 10.3390/molecules29235590 (PMC11643398; doi:10.3390/molecules29235590)
Supplement: Supplementary file 1 [file molecules-29-05590-s001.zip › molecules-3148181-supplementary.pdf]

Supporting information

Preparation and mechanical behavior of metal-rubber assemblies assisted by plasma treatment

Lazhar Benyahia *, Marisol Ji and Fabienne Poncin-Epaillard *

Institut des Molécules et Matériaux du Mans, IMMM, UMR CNRS n° 6283, Le Mans Université, Avenue Olivier Messiaen, 72085 Le Mans, France.

* Corresponding authors:

E-mail address: Lazhar.Benyahia@univ-lemans.fr; Fabienne.poncin-epaillard@univ-lemans.fr

Table S1: Young (tack) and Young (rheology) moduli for Al–pp–NBR for the different plasma deposits

	E (MPa)				E_{rheo} (MPa)
	PW	CW	CW x 2	CW + PW	
pp–Ac	17.3<br>12.3<br>13.7	14.4<br>20.7<br>10.8	21.9<br>11.5<br>15.0	16.2<br>17.7<br>17.3	10.6 ± 0.05
pp–AA	5.9<br>3.5<br>6.8	4.8<br>5.2<br>5.8	6.2<br>6.2<br>1.1	5.3<br>5.4<br>–	10.7 ± 0.05
pp–MA	11.6<br>10.4<br>12.6<br>-	17.5<br>8.4<br>21.9<br>-	11.1<br>16.9<br>14.2<br>14.0	16.8<br>16.3<br>21.8<br>-	9.90 ± 0.02

Table S2: Young (tack) and Young (rheology) moduli for SS–pp–FKM for the different plasma deposits

	E (MPa)				E_{rheo} (MPa)
	PW	CW	CW x 2	CW + PW	
pp–Ac	8.8<br>8.5<br>9.4	9.3<br>9.2<br>6.1	9.2<br>18.7<br>18.5	9.6<br>3.1<br>3.4	11.5 ± 0.04
pp–AA	18.3<br>17.9<br>18.3	17.8<br>18.4<br>17.4	18.4<br>18.7<br>21.9	19.0<br>18.9<br>18.7	11.7 ± 0.01
pp–MA	21.4<br>19.1<br>21.2	19.5<br>24.2<br>18.5	17.7<br>18.9<br>17.2	21.8<br>18.0<br>23.2	11.8 ± 0.02

Table S3: Wavenumber area of the different FTIR vibration bands

	C=C band	C–H band	C=O band
Ac	1576 - 1695 cm^{-1}	1320 - 1489 cm^{-1}	1626 - 1770-cm^{-1}
AA	1507 - 1741 cm^{-1}	1316 - 1490 cm^{-1}	1635 - 1813 cm^{-1}
MA	1564 - 1715 cm^{-1}	1326 - 1493 cm^{-1}	1626 - 1824 cm^{-1}

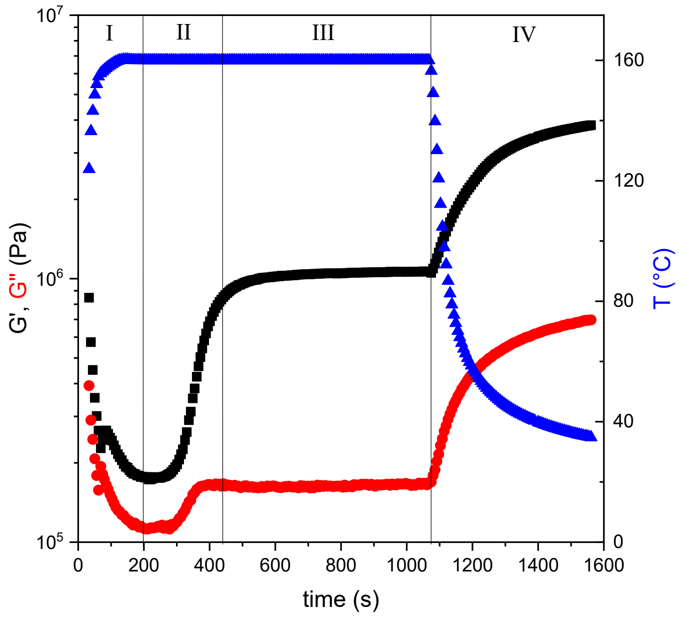

Figure S1: Time dependence of storage viscoelastic modulus G' (black scares), loss viscoelastic modulus G'' (red circles) and temperature (blue triangles) during curing time for an Al–NBR assembly without plasma deposition.

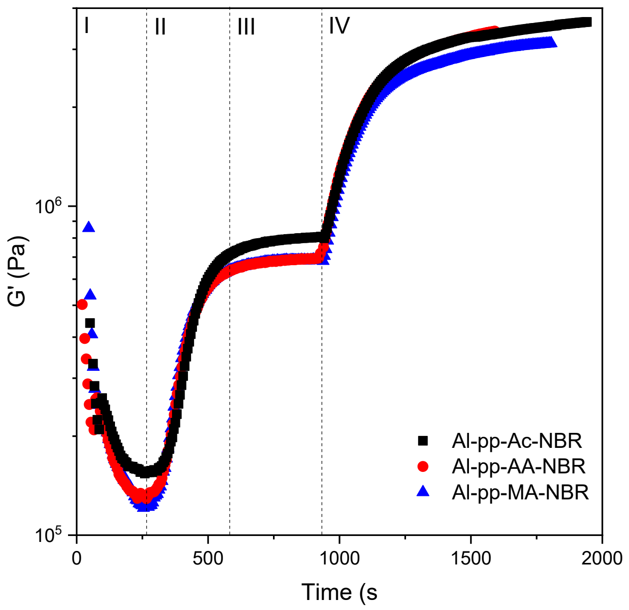

Figure S2: Time–dependence of the elastic modulus G' during vulcanization for different Al–pp–NBR assemblies treated with a pulsed plasma (PW) with different precursors. Zone I corresponds to the softening of the elastomer at the beginning of heating, Zone II illustrates the initiation of crosslinking, Zone III is the accomplishment of the vulcanization during the isotherm stage. Zone IV corresponds to the cooling of the sample.

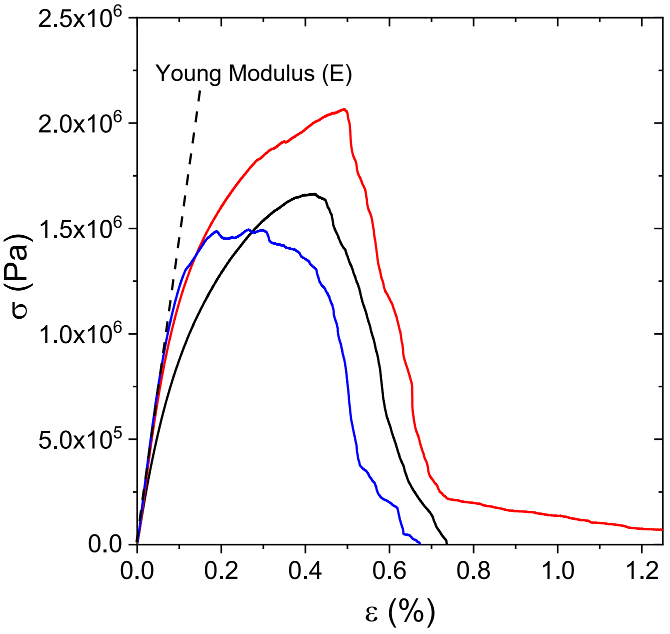

Figure S3: Typical tensile curves showing the nominal stress σ versus the true strain ε for 3 Al–ppMA (PW)–NBR assemblies. The dashed line represent the linear fir of the beginning of the curve corresponding to the linear regime. The slop of this lie represents the Young modulus E

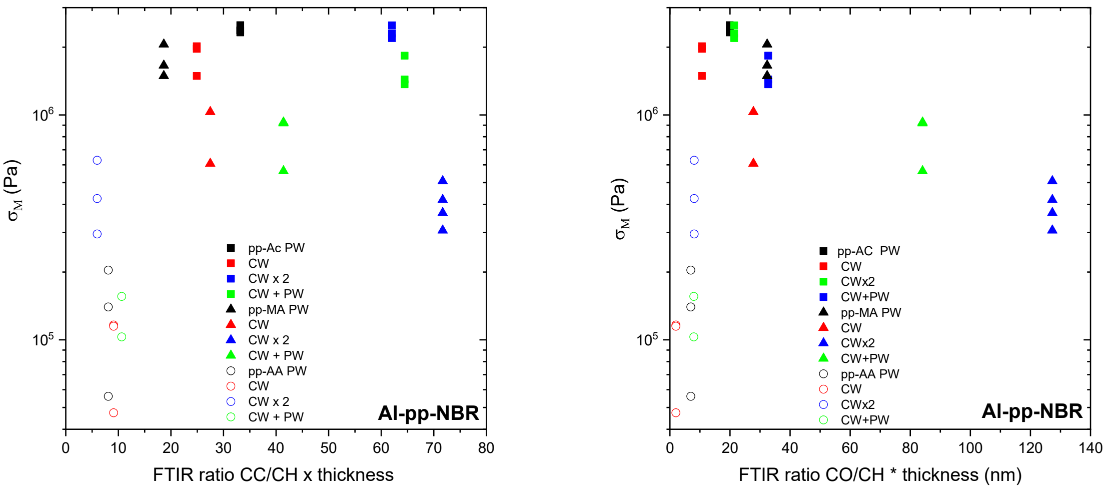

Figure S4: Dependence of the maximal stress σ_M on the chemistry determined by FTIR spectroscopy for Al–pp–NBR assemblies. Empty symbols correspond to assemblies for which E is lower than E_{rheo}.

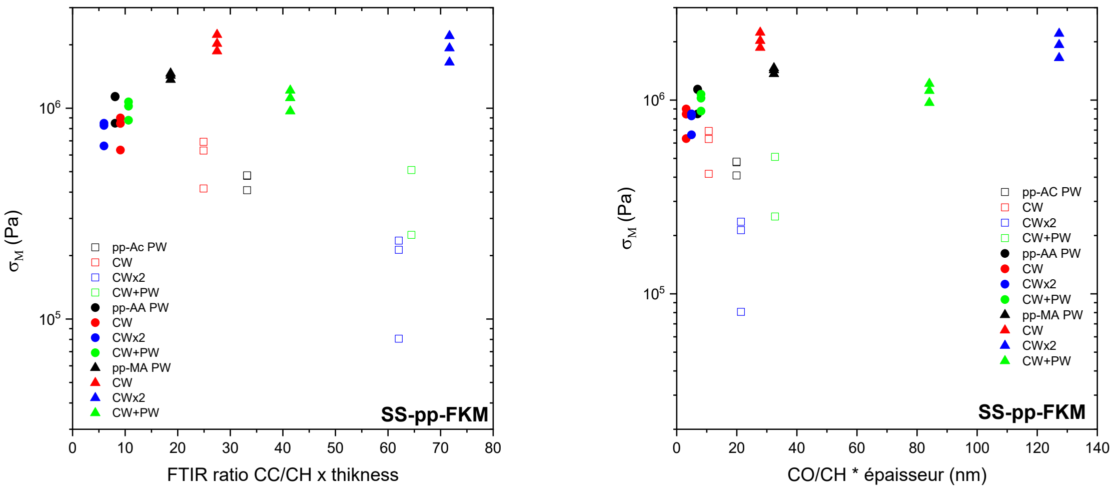

Figure S5: Dependence of the maximal stress σ_M on the chemistry determined by FTIR spectroscopy for SS–pp–FKM assemblies. Empty symbols correspond to assemblies for which E is lower than E_{rheo}.
